# Manual Intermittent Positive Airway Pressure (IPAP) for the Management of Secretion-Related Hypoxemia During One-Lung Ventilation: A Case Report

**DOI:** 10.7759/cureus.106647

**Published:** 2026-04-08

**Authors:** Ökkeş Hakan Miniksar, Seher Altınel, Hakan Nomenoğlu, Nurcan Özaslan, Mustafa Kalkan, Göktürk Fındık

**Affiliations:** 1 Anesthesiology and Reanimation, Dr. Abdurrahman Yurtaslan Ankara Oncology Training and Research Hospital, Ankara, TUR; 2 Thoracic Surgery, Dr. Abdurrahman Yurtaslan Ankara Oncology Training and Research Hospital, Ankara, TUR

**Keywords:** fiberoptic bronchoscopy, hypoxemia management, intermittent positive airway pressure, one-lung ventilation (olv), thoracic anesthesia

## Abstract

Hypoxemia during one-lung ventilation (OLV) is a well-established complication in thoracic surgery, typically resulting from an increased intrapulmonary shunt due to continued perfusion of the non-ventilated lung. However, some episodes cannot be attributed solely to shunt-related mechanisms, as airway obstruction from excessive secretions in the ventilated lung may also cause impaired ventilation, severe hypercapnia, and reduced lung compliance. We report the case of a 64-year-old male patient undergoing left-sided video-assisted thoracoscopic surgery in whom severe hypoxemia and hypercapnia developed shortly after the initiation of OLV. Fiberoptic bronchoscopy revealed dense purulent secretions in the ventilated lung, and standard rescue strategies failed to restore adequate oxygenation. Manual intermittent positive airway pressure (IPAP) was therefore applied to the non-ventilated lung while bronchoscopic aspiration and lavage were performed. Following the application of manual IPAP, oxygen saturation improved, PaCO₂ decreased markedly, and lung compliance increased, allowing the surgery to be completed without further complications. This case suggests that hypoxemia during OLV may result from mechanisms other than an increased intrapulmonary shunt and that manual IPAP applied to the non-ventilated lung may represent an effective rescue strategy in cases associated with ventilation impairment due to secretion accumulation.

## Introduction

One-lung ventilation (OLV) is a commonly used technique in thoracic surgery in which one lung is selectively ventilated while the other is intentionally collapsed to facilitate surgical exposure [1]. Hypoxemia during OLV is a well-recognized complication, most commonly attributed to an increased intrapulmonary shunt from continued perfusion of the non-ventilated lung. However, some hypoxemic events cannot be explained solely by shunt physiology [1,2]. Airway obstruction caused by excessive secretion accumulation in the ventilated (dependent) lung may impair alveolar ventilation, leading to carbon dioxide retention, decreased lung compliance, and secondary hypoxemia [1-7].

The application of intermittent positive airway pressure (IPAP) to the non-ventilated lung was first described by Russell as a potential rescue maneuver [4]. However, limited evidence exists regarding its use in ventilation impairment related to secretion accumulation. In this report, we present a case in which acute hypoxemia and severe hypercapnia developing during OLV were successfully managed using manual IPAP.

## Case presentation

A 64-year-old male patient, 187 cm in height and weighing 100 kg, was scheduled for left upper lobectomy via left-sided video-assisted thoracoscopic surgery due to a malignant lesion in the left upper lobe. The patient had no known chronic medical conditions or prior surgical history, except for a history of cigarette smoking. Preoperative pulmonary function tests revealed an FEV₁ of 3.5 L (95% predicted) and an FEV₁/FVC ratio of 79% (105% predicted).

Upon admission to the operating room, blood pressure was 130/79 mmHg, heart rate was 77 beats/min, and SpO₂ was 98%. Standard American Society of Anesthesiologists monitoring and bispectral index monitoring were applied. For postoperative analgesia, a thoracic epidural catheter was inserted aseptically at the T5-T6 level. Following preoxygenation, anesthesia was induced with fentanyl 100 µg, lidocaine 100 mg, propofol 200 mg, and rocuronium 50 mg. Intubation was performed using a 41 Fr left-sided double-lumen tube (DLT), and tube position was confirmed by fiberoptic bronchoscopy (FOB). Anesthesia was maintained with total intravenous anesthesia using propofol (4-6 mg/kg/h) and remifentanil (0.05-0.2 µg/kg/min). Invasive arterial blood pressure monitoring was established via a 20-G arterial catheter placed in the right radial artery.

After positioning the patient in the right lateral decubitus position, OLV was initiated, and the surgical procedure began. During the first 10 minutes, SpO₂ remained stable between 95% and 100%. However, sudden desaturation developed, and SpO₂ decreased to approximately 70%. Tidal volume dropped to 100 mL, peak airway pressure was 30 cmH₂O, plateau pressure was 28 cmH₂O, and lung compliance decreased to 25 mL/cmH₂O. To manage hypoxemia, FiO₂ was increased to 1.0, a recruitment maneuver was performed, and PEEP was subsequently optimized (from 5 to 8 cmH₂O). Despite these interventions, SpO₂ fluctuated between 70% and 94%. Following FOB evaluation, two-lung ventilation was resumed, and SpO₂ increased to 96%. Arterial blood gas analysis obtained approximately 10-15 minutes after these interventions revealed a pH of 7.10, PaO₂ of 97 mmHg, and PaCO₂ of 90 mmHg.

The position of the DLT was reconfirmed using FOB, and dense purulent secretions were observed in the ventilated lung and aspirated. However, during bronchoscopic aspiration of the ventilated lung, the patient developed recurrent rapid desaturation. OLV was reinitiated; however, adequate ventilation could not be achieved, and desaturation persisted. Therefore, manual IPAP was applied to the non-ventilated lung while segmental bronchoalveolar lavage and aspiration were performed under FOB guidance.

**Figure 1:**
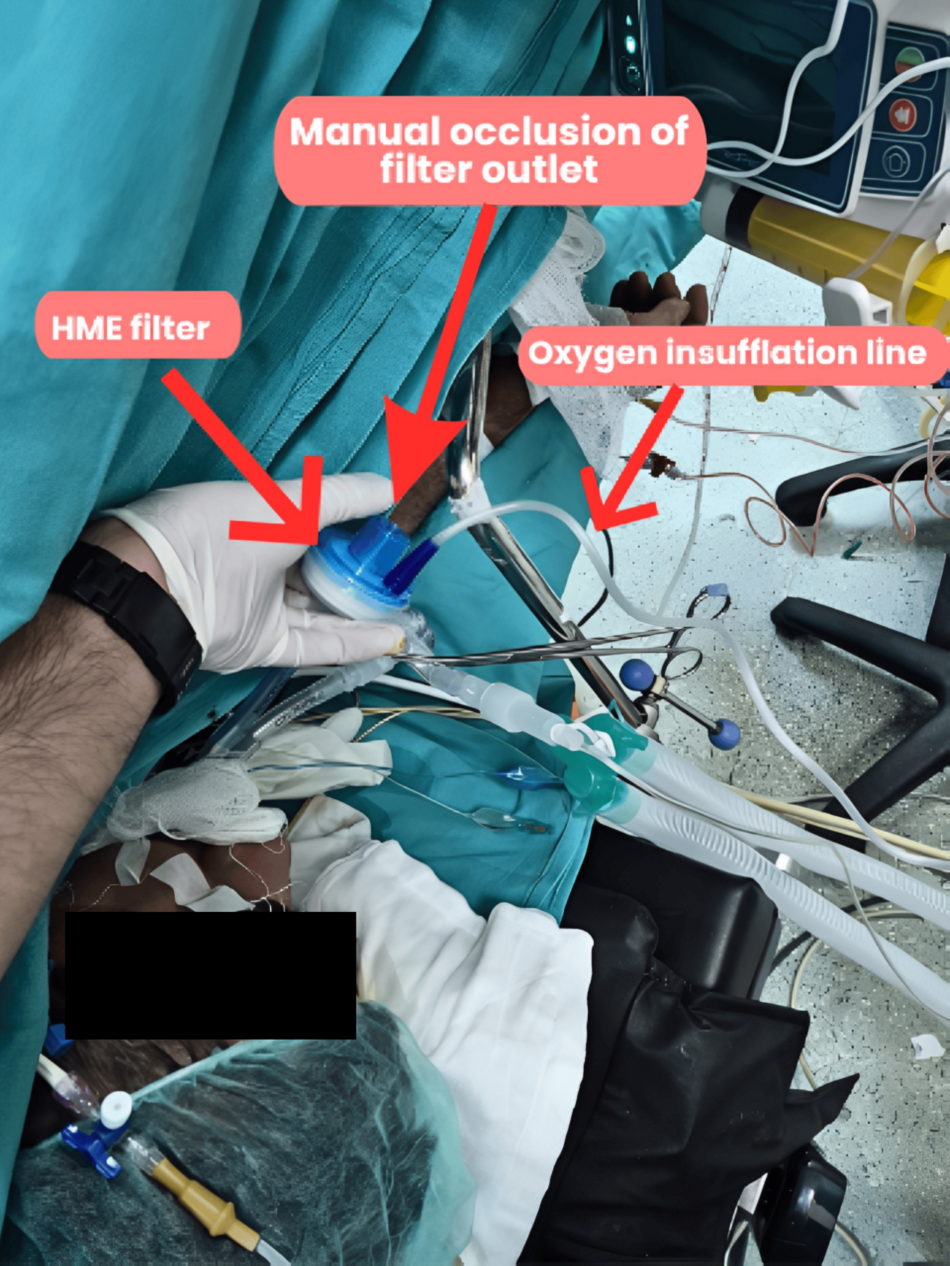
Configuration of the manual IPAP technique showing the oxygen insufflation line connected to the gas sampling port of the HME filter attached to the DLT Y-connector DLT, double-lumen tube; HME, heat and moisture exchanger; IPAP, intermittent positive airway pressure

The manual IPAP technique was applied as follows: a heat and moisture exchanger (HME) filter was placed at the Y-connector of the DLT, and the oxygen tubing from the anesthesia machine was connected to the gas sampling port of the filter with the flow set at 4 L/min (66 mL/s) (Figure 1). The open port of the filter was occluded for two seconds to deliver approximately 132 mL of oxygen into the non-ventilated lung, followed by a four-second release to allow passive deflation. Using six-second cycles (10 cycles/min), approximately 132 mL of oxygen was delivered per cycle, corresponding to a total oxygen inflation of approximately 1320 mL/min (Video 1).

**Video 1:** Demonstration of the manual IPAP technique applied to the non-ventilated lung during OLV IPAP, intermittent positive airway pressure; OLV, one-lung ventilation

Ten minutes after the initiation of manual IPAP, SpO₂ increased to 95%, PaCO₂ decreased to 51 mmHg, and lung compliance improved to 38 mL/cmH₂O. The duration of OLV was 180 minutes, and the total surgical time was 360 minutes. As demonstrated in Video 1, low-volume ventilation of the non-ventilated lung using manual IPAP did not interfere with the surgical field, and surgical satisfaction was not compromised. The procedure was extended to a pneumonectomy after an intraoperative frozen section revealed malignancy in an additional lower lobe nodule. During surgery, urine output was 1100 mL, 3500 mL of intravenous fluids were administered, and approximately 300 mL of blood loss occurred. The patient was successfully extubated in the operating room and transferred to the intensive care unit for postoperative monitoring.

## Discussion

Hypoxemia during OLV is most commonly explained by an increase in intrapulmonary shunt resulting from continued perfusion of the non-ventilated lung [1]. The lateral decubitus position, alveolar collapse, and ventilation-perfusion (V/Q) mismatch further exacerbate this pathophysiology and negatively affect gas exchange [1,2]. Therefore, standard management strategies for hypoxemia during OLV include increasing FiO₂, confirming the position of the DLT, optimizing PEEP in the ventilated lung, applying CPAP or small tidal volume ventilation to the non-ventilated lung, administering apneic oxygen insufflation, and, when necessary, reverting to two-lung ventilation [1,3-6].

In recent years, several studies have supported the use of ventilation strategies targeting the non-ventilated lung. These studies have demonstrated that apneic oxygen insufflation reduces the incidence of hypoxemia during thoracic surgery [6-9], high-flow oxygen administration may improve oxygenation compared with conventional CPAP [3], and the combination of CPAP with small tidal volume ventilation can increase arterial oxygenation and reduce the pulmonary shunt fraction [5]. The common principle underlying these approaches is the reduction of shunt fraction by increasing alveolar oxygen partial pressure in the non-ventilated lung through continuous or semi-continuous positive pressure.

However, not all hypoxemic episodes during OLV can be explained solely by increased intrapulmonary shunt. In the present case, severe hypoxemia was accompanied by marked hypercapnia (PaCO₂ 90 mmHg) and a sudden decrease in lung compliance. These findings suggested that the primary mechanism was not only shunt physiology but also a significant loss of ventilation. Despite the application of most rescue maneuvers recommended in the literature, adequate oxygenation could not be achieved. Consequently, the IPAP technique, first described by Russell [4] and applied in our clinical practice, was implemented, resulting in successful resolution of hypoxemia. The application of CPAP or oxygen insufflation to the non-ventilated lung may interfere with the surgical field and is therefore considered disadvantageous.

Previous studies have suggested that ventilation impairment due to secretion accumulation may represent an important mechanism, particularly in prolonged OLV procedures [2,7-9]. In the present case, the marked reduction in ventilation and the development of hypoxemia were explained by dense purulent secretions observed in the ventilated lung during bronchoscopy. The presence of these secretions may be related to smoking-associated airway inflammation or a possible subclinical respiratory infection, although the exact etiology could not be definitively determined.

Apneic oxygen insufflation and high-flow oxygen therapy are primarily diffusion-based, passive oxygenation strategies [3,6]. Although HFJV can be used to improve oxygenation in similar situations, its requirement for specialized equipment and expertise may limit its immediate applicability. In contrast, manual IPAP is a simple, readily available, and practical maneuver that can be performed without additional devices. CPAP maintains alveolar patency, thereby reducing shunt and improving oxygenation [5]. In contrast, IPAP provides active ventilatory support through intermittent inflation-deflation cycles, improving both oxygenation and carbon dioxide elimination. For this reason, IPAP may represent a physiologically more rational approach in hypoxemic states associated with secretion accumulation or ventilation loss. In addition, the absence of significant impairment in the surgical field in this case supports the practical feasibility of this technique.

## Conclusions

Hypoxemia during OLV is a multifactorial condition that cannot be explained solely by intrapulmonary shunt. In selected cases with ventilation impairment and hypercapnia, manual IPAP applied to the non-ventilated lung may serve as an effective rescue strategy. However, as this report is based on a single case, the findings should be interpreted with caution, and further prospective studies are needed to confirm its efficacy and safety.

## References

[1] Lohser J, Slinger P (2015). Lung injury after one-lung ventilation: a review of the pathophysiologic mechanisms affecting the ventilated and the collapsed lung. Anesth Analg.

[2] Slinger P, Campos JH (2015). Anesthesia for thoracic surgery. Miller’s Anesthesia, Eighth Edition.

[3] Sawasdiwipachai P, Weerayutwattana R, Thongcharoen P, Suksompong S (2021). Comparison of high-flow humidified oxygen with conventional continuous positive airway pressure in nonventilated lungs during thoracic surgery: a randomized cross-over study. J Cardiothorac Vasc Anesth.

[4] Russell WJ (2009). Intermittent positive airway pressure to manage hypoxia during one-lung anaesthesia. Anaesth Intensive Care.

[5] Yang Y, Jia D, Cheng L, Jia K, Wang J (2024). Continuous positive airway pressure combined with small-tidal-volume ventilation on arterial oxygenation and pulmonary shunt during one-lung ventilation in patients undergoing video-assisted thoracoscopic lobectomy: a randomized, controlled study. Ann Thorac Med.

[6] Jung DM, Ahn HJ, Jung SH, Yang M, Kim JA, Shin SM, Jeon S (2017). Apneic oxygen insufflation decreases the incidence of hypoxemia during one-lung ventilation in open and thoracoscopic pulmonary lobectomy: a randomized controlled trial. J Thorac Cardiovasc Surg.

[7] Karzai W, Schwarzkopf K (2009). Hypoxemia during one-lung ventilation: prediction, prevention, and treatment. Anesthesiology.

[8] Campos JH (2002). Current techniques for perioperative lung isolation in adults. Anesthesiology.

[9] Duggan M, Kavanagh BP (2005). Pulmonary atelectasis: a pathogenic perioperative entity. Anesthesiology.

